# Professional Secrecy and Syphilis

**Published:** 1907-10

**Authors:** E. S. Mckee

**Affiliations:** Cincinnati


### Professional Secrecy and Syphilis.

E. S. MCKEE, M. D., Cincinnati.

This was the subject of an address by Professor Parisot on taking his seat as President of the Société de Medicine de Nantes. He took up the discussion of French practitioners in relation to article 378 of the penal code, which forbids the disclosure of professional secrets except in cases where the law requires it. In 1892 the law was set aside so far as to allow of the notification of professional diseases, epidemic in character. No professional prejudice ought to be allowed to obstruct a law which is eminently useful, but if it is desirable to notify mumps and measles on account of their contagiousness and danger to the public good, why not include syphilis? True it is that it would be difficult to get medical men to report these cases, probably more difficult than to get them to report the births of illegitimate children, and there would be the danger of patients refusing to consult a doctor in such cases if it entailed publicity. We have, in fact, many persons now who refuse to call a physician in mumps or measles to obviate the restrictions resulting from being reported under the contagious disease act. He had even noted this occurring in patients suffering from so serious a disease as smallpox. Effective measures should be taken to prevent anyone suffering from syphilis from communicating it to innocent persons. Parisot maintains, justly, that one who would knowingly communicate this terrible malady is a criminal who should be made to suffer the extreme penalties of the law. If parents engage a healthy wet nurse for their syphilitic child it is the duty of the medical attendant to warn her of the danger without naming the disease. Neglect to do this renders him liable, as the instance cited in the court of Dijon, May 14, 1868.

Parisot is of the opinion that where the practitioner is called by the employer to prescribe for a servant and discovers her to be suffering from syphilis, he should give her a written diagnosis, with directions what to do and what to abstain from in order to prevent risk from infection, and a prescription for the necessary remedies; then, if asked for his opinion as to the nature of the case, he can refer the employers to the servant, stating that he has given his opinion to her in writing, and that she is the proper person to inform them if she sees fit.

One of the most difficult and trying questions which comes to torment the medical man is that of the patient who, in spite of solemn warnings, insists on getting married. It is best to stop such inquiries in the onset. We should never discuss with a third party the case of a patient requiring discretion without the patient's permission. Of course, the rigor of the law is often deplorable and goes directly contrary to one's conscience. It is to be hoped that there will some day be legislation for the control of syphilis, and the hope of the total eradication of all contagious diseases will be realised. The opinions of old practitioners who have practised for many years in the same locality and observed the dangers of the present system over two or three generations of families ought to be of value on this subject.

